# A Pre-Operative Prognostic Score for Patients With Advanced Hepatocellular Carcinoma Who Underwent Resection

**DOI:** 10.3389/fonc.2021.569515

**Published:** 2021-02-26

**Authors:** Han Xiao, Jia-Li Li, Shu-Ling Chen, Mi-Mi Tang, Qian Zhou, Ting-Fan Wu, Xin Li, Zhen-Wei Peng, Shi-Ting Feng, Sui Peng, Ming Kuang

**Affiliations:** ^1^ Division of Interventional Ultrasound, The First Affiliated Hospital of Sun Yat-sen University, Guangzhou, China; ^2^ Department of Liver Surgery, Dongguan People’s Hospital, Dongguan, China; ^3^ Department of Medical Imaging, The First Affiliated Hospital of Sun Yat-sen University, Guangzhou, China; ^4^ Clinical Trials Unit, The First Affiliated Hospital of Sun Yat-sen University, Guangzhou, China; ^5^ Clinical Education Team, GE Healthcare, Beijing, China; ^6^ Global Research, GE Healthcare, Beijing, China; ^7^ Department of Radiotherapy, The First Affiliated Hospital of Sun Yat-sen University, Guangzhou, China; ^8^ Department of Gastroenterology, The First Affiliated Hospital of Sun Yat-sen University, Guangzhou, China; ^9^ Department of Liver Surgery, The First Affiliated Hospital of Sun Yat-sen University, Guangzhou, China

**Keywords:** hepatocellular carcinoma, tumor thrombosis, macrovascular invasion, surgery, prognostic score

## Abstract

**Background:**

Previous studies demonstrated a promising prognosis in advanced hepatocellular carcinoma (HCC) patients who underwent surgery, yet a consensus of which population would benefit most from surgery is still unreached.

**Method:**

A total of 496 advanced HCC patients who initially underwent liver resection were consecutively collected. Least absolute shrinkage and selection operator (LASSO) regression was performed to select significant pre-operative factors for recurrence-free survival (RFS). A prognostic score constructed from these factors was used to divide patients into different risk groups. Survivals were compared between groups with log-rank test. The area under curves (AUC) of the time-dependent receiver operating characteristics was used to evaluate the predictive accuracy of prognostic score.

**Result:**

For the entire cohort, the median overall survival (OS) was 23.0 months and the median RFS was 12.1 months. Patients were divided into two risk groups according to the prognostic score constructed with ALBI score, tumor size, tumor-invaded liver segments, gamma-glutamyl transpeptidase, alpha fetoprotein, and portal vein tumor thrombus stage. The median RFS of the low-risk group was significantly longer than that of the high-risk group in both the training (10.1 vs 2.9 months, *P*<0.001) and the validation groups (13.7 vs 4.6 months, *P*=0.002). The AUCs of the prognostic score in predicting survival were 0.70 to 0.71 in the training group and 0.71 to 0.72 in the validation group.

**Conclusion:**

Surgery could provide promising survival for HCC patients at an advanced stage. Our developed pre-operative prognostic score is effective in identifying advanced-stage HCC patients with better survival benefit for surgery.

## Introduction

Yearly, about 365 thousand new cases of hepatocellular carcinoma (HCC) patients are diagnosed in China, and 319 thousand patients die of HCC (1, 2). The high morbidity and mortality make HCC a huge disease burden in China. Accordingly, over 50% of HCC patients are in an advanced stage at the first diagnosis (3). Patients at an advanced stage usually have a poor prognosis, especially those accompanied with macrovascular invasion.

No treatment was proven to be effective for advanced HCC patients until a large randomized clinical trial (RCT) claimed that sorafenib could prolong the overall survival (OS) of HCC patients at an advanced stage in 2008 (4). Later, lenvatinib was also introduced into the first-line treatment in 2018 (5). However, the median OS was only 8.1-9.8 months for patients with macrovascular invasion, which was far from satisfactory.

Over the past decade, lots of effort has been made to search for other approaches to improve the survival of advanced HCC patients. Resection is the most frequently applied curative treatment of HCC and is also generally performed among advanced-stage HCC patients in real clinical practice (3, 6). Patients were about three times more likely to receive resection than sorafenib (6). Evidence also showed that advanced HCC patients who underwent resection could have a significantly better survival than those in the non-resection group (7, 8). This was especially true for patients with macrovascular invasion, where the median survival could be prolonged by 1.77 years in the resection group if the patients had Child-pugh A stage liver function (9). Despite the reported survival benefits, the recommendation of surgery in advanced HCC is quite controversial. The guidelines of the American Association for the Study of Liver Diseases (AASLD) and the European Association for the Study of the Liver (EASL) took advanced HCC as a contradiction to surgery (10, 11). However, the guidelines of the Asian Pacific Association for the Study of the Liver (APASL) and the Japan Society of Hepatology (JHS) agreed that resection could be performed in some advanced HCC patients (12, 13). The differences between guidelines might be due to the huge heterogeneity within advanced HCC. Although some advanced HCC patients could reach a median survival of more than 4 years, others might have a similar survival with sorafenib treatment but still experience an invasive treatment procedure (9, 14–17). Therefore, finding out a super-selection of the population who might benefit most from surgery pre-operatively would help in proper treatment selection for advanced HCC patients.

Many factors influence the survival of advanced HCC patients, including tumor number, cancer cell differentiation, etc. (18). One of the most important prognostic factors is the stage of macrovascular invasion, especially the portal vein tumor thrombosis (PVTT). A study including 2093 advanced HCC patients showed that median survival could range from 0.91 to 4.13 years due to the different stages of PVTT (Vp4 to Vp1) (9). The stage of PVTT also influences the type and extent of surgery. Therefore, taking the stage of PVTT into consideration is necessary for the management of advanced-stage HCC patients. An EHBH-PVTT scoring system was established recently using four elements to predict survival in advanced HCC patients (19). However, the stage of PVTT was described in this study instead of being selected as a prognostic factor. To our knowledge, none of the published studies have evaluated the prognostic effect of the stage of PVTT.

Therefore, this article retrospectively analyzed HCC patients at an advanced stage who underwent resection, aiming to establish a prognostic score based on the stage of PVTT and other pre-operative clinical factors and to give some evidence on proper candidate selection for resection in advanced HCC.

## Method

### Patients Selection

This is a retrospective study based on a prospectively collected database from the First Affiliated Hospital of Sun Yat-sen University. From May 30^th^, 1995 to June 1^st^, 2017, 3 168 HCC patients who initially underwent liver resection were consecutively collected. HCC were diagnosed following the guidelines of the time (20–22). The inclusion criteria were as follows: a) primary HCC without previous treatment, b) Barcelona Clinic Liver Cancer (BCLC) stage C (23), c) Child-pugh stage A-B, and d) Eastern Cooperative Oncology Group (ECOG) grades 0-1. The exclusion criteria were: a) extrahepatic metastasis, b) patients who underwent palliative resection, or c) data of enhanced computed tomography (CT) or magnetic resonance (MR) not available. Eventually, 496 patients were included in this study.

### Data Collection

At least 1 instance of enhanced CT or MR was performed within 1 month before resection for each patient. Tumor size, tumor number, and the stage of PVTT and hepatic vein tumor thrombus (HVTT) were evaluated on CT or MR, by two radiologists with over 5 years of experience. The stage of PVTT and HVTT were defined according to the stage system in Japan (18). PVTT were categorized as PVTT 4 (portal invasion at the main portal trunk), PVTT 3 (portal invasion at the first order branch), PVTT 2 (portal invasion at the second order branch), PVTT 1 (portal invasion at the third or more peripheral branch), and PVTT 0 (absence of portal invasion). HVTT were categorized as HVTT 3 (tumor thrombosis in the inferior vena cava), HVTT 2 (tumor thrombosis in a main hepatic vein), HVTT 1 (tumor thrombosis in a peripheral hepatic vein), and HVTT 0 (absence of HVTT). Patient characteristics including age, gender, and ECOG performance status at the time of surgery were collected. Latest results of laboratory tests, including levels of alpha fetoprotein (AFP), platelet (PLT), hemoglobin (HB), albumin (ALB), total bilirubin (TB), alanine transaminase (ALT), aspartate aminotransferase (AST), gamma-glutamyl transpeptidase (GGT), and prothrombin time (PT), were collected before surgery. Status of ascites, splenomegaly, and varicosity were also included in the analysis. Portal hypertension (PHT) was defined as esophageal varices or splenomegaly associated with a platelet count lower than 100×10^9^/L (24). The BCLC stage and Child-pugh grade were derived based on the radiology and laboratory findings.

### Treatment and Follow-Up

Surgery was performed under general anesthesia by surgeons with 10–40 years of experience. The type of surgery was decided according to a routine discussion for each patient in the Department of Liver Surgery. Anatomic or non-anatomic resection was decided according to the tumor burden and liver function of the patients. The surgical approach was chosen based on the liver remnant, tumor location, and preference of the operator. Intraoperative ultrasound (US) was used to assist in operative evaluation.

Evaluation of recurrence was performed at the first month after initial resection and was repeated every 3 months for the first two years, and 3-6 months thereafter. Either US or CT was performed for evaluation during the follow-up. Once a focal in liver was detected by the US, the patient would receive CT or MR for further diagnosis. Recurrence was evaluated according to the criterion of HCC diagnosis in the EASL guideline. Treatment recommendations for recurrent HCC was made by the physician after evaluation of the tumor burden, liver function, and patient’s common status. Curative treatments were recommended if possible, and the final decision was made by the patient.

### Statistical Analysis

Patients were randomly divided into the training group (n=347) and the validation group (n=149) by a ratio of 7:3. Normal distribution test was performed for continuous variables. Continuous variables that obey normal distribution were presented as means ± SD and others as median and quartile. Categorical variables were presented as numbers and percentages. Differences between the training group and validation group were compared with the t-test for continuous variables and χ^2^ test for categorical variables.

Recurrence-free survival (RFS) was defined as the time interval from the date of surgery to the date of first radiology confirmed recurrence according to the modified response criteria in solid tumors (25), the date of death from any cause, or to the date of the last follow-up visit. Overall survival (OS) was defined as the time interval from the date of surgery to the date of death from any cause or to the date of the last follow-up visit. The predicting model for RFS was constructed with the data of the training group. Least absolute shrinkage and selection operator (LASSO) regression followed by a stepwise analysis were performed to select factors to build the model. Time dependent Receiver Operating Characteristic (ROC) curve was used to calculate the area under the curve (AUC) for the evaluation of the model in both the training and the validation group. The cutoff was set to achieve the highest accuracy in the training group and was then applied to the validation group. Patients would be divided into the high-risk group and the low-risk group with this cutoff. Survival curves of RFS and OS in different risk groups were generated by the Kaplan–Meier method and compared by the log-rank test. Subgroup analyses were also performed according to whether the patients had the condition of ascites or PHT or thrombocytopenia or in Child-Pugh class B. Statistical significance was considered as a two-sided P value of less than 0.05. The above statistical analysis was performed with the STATA/MP 14.0.

## Result

### Patient Characteristics

Baseline characteristics of the training and validation groups were shown in **Table 1**. After excluding 85 patients with palliative resection, a total of 496 patients were enrolled. The majority of patients with HVTT were in HVTT 2 stage. As for the category of PVTT, only 17 patients had PVTT in the main trunk (3.4%). PVTT 2 and 3 stage accounted for 31.0% and 25.0% of all patients, respectively. Only 3.7% of the patients had a history of HCV affected, and 86.7% of the patients were HBsAg-positive. The majority of patients were in Child-Pugh stage A (90.1%). And most of the patients had tumors larger than 5cm (85.3%). The pre-treatment characteristics were similar between the training group and the validation group, except that the ratio of multiple lesions was higher in the training group (40.6% vs 29.5%, *P*=0.019).

**Table 1:** Baseline characteristics of the training group and the validation group.

Variables		Total	Training	Validation	P value
Age (years)	≤65	434(87.5%)	305(87.9%)	129(86.6%)	0.684
	>65	62(12.5%)	42(12.1%)	20(13.4%)	
Tumor size (cm)	<=5	73(14.7%)	52(15.0%)	21(14.1%)	0.797
	>5	423(85.3%)	295(85.0%)	128(85.9%)	
	Median (IQR)	9.6 (6.5, 12.7)	9.6 (6.5, 12.9)	9.4 (6.4, 12.0)	0.552
Splenomegaly	No	275(55.4%)	187(53.9%)	88(59.1%)	0.288
	Yes	221(44.6%)	160(46.1%)	61(40.9%)	
Ascites	No	448(90.3%)	309(89.0%)	139(93.3%)	0.143
	Yes	48(9.7%)	38(11.0%)	10(6.7%)	
Child-Pugh stage	A	447(90.1%)	312(89.9%)	135(90.6%)	0.813
	B	49(9.9%)	35(10.1%)	14(9.4%)	
ALBI score	≤-2.6	203(40.9%)	140(40.3%)	63(42.3%)	0.688
	>-2.6	293(59.1%)	207(59.7%)	86(57.7%)	
Tumor number	Single	311(62.7%)	206(59.4%)	105(70.5%)	0.019
	Multiple	185(37.3%)	141(40.6%)	44(29.5%)	
	Median (IQR)	1.0 (1.0, 3.0)	1.0 (1.0, 4.0)	1.0 (1.0, 2.0)	0.003
PHT	No	424(85.5%)	296(85.3%)	128(85.9%)	0.861
	Yes	72(14.5%)	51(14.7%)	21(14.1%)	
AFP (ng/mL)	≤200	190(38.3%)	141(40.6%)	49(32.9%)	0.104
	>200	306(61.7%)	206(59.4%)	100(67.1%)	
PVTT class	0-2	355(71.6%)	252(72.6%)	103(69.1%)	0.429
	3-4	141(28.4%)	95(27.4%)	46(30.9%)	
HVTT class	0-1	359(72.4%)	251(72.3%)	108(72.5%)	0.973
	2-3	137(27.6%)	96(27.7%)	41(27.5%)	
NLR	≤2.2	206(41.5%)	143(41.2%)	63(42.3%)	0.824
	>2.2	290(58.5%)	204(58.8%)	86(57.7%)	
PLT (10^9^/L)	≤100	29(5.8%)	18(5.2%)	11(7.4%)	0.339
	>100	467(94.2%)	329(94.8%)	138(92.6%)	
ALB (g/L)	>35	375(75.6%)	265(76.4%)	110(73.8%)	0.545
	≤35	121(24.4%)	82(23.6%)	39(26.2%)	
TB (umol/L)	≤34.2	464(93.5%)	324(93.4%)	140(94.0%)	0.807
	>34.2	32(6.5%)	23(6.6%)	9(6.0%)	
ALT (U/L)	≤40	241(48.6%)	166(47.8%)	75(50.3%)	0.610
	>40	255(51.4%)	181(52.2%)	74(49.7%)	
AST (U/L)	≤40	179(36.1%)	124(35.7%)	55(36.9%)	0.802
	>40	317(63.9%)	223(64.3%)	94(63.1%)	
GGT (U/L)	≤50	72(14.5%)	49(14.1%)	23(15.4%)	0.703
	>50	424(85.5%)	298(85.9%)	126(84.6%)	
HB (g/L)	≤120	107(21.6%)	81(23.3%)	26(17.4%)	0.144
	>120	389(78.4%)	266(76.7%)	123(82.6%)	
HBsAg	missing	3(0.6%)	1(0.3%)	2(1.3%)	0.258
	negative	63(12.7%)	42(12.1%)	21(14.1%)	
	positive	430(86.7%)	304(87.6%)	126(84.6%)	

IQR, interquartile range; PHT, portal hypertension; AFP, alpha fetoprotein; HVTT, hepatic vein tumor thrombosis; PVTT, portal vein tumor thrombosis; NLR, neutrophil to lymphocyte ratio; PLT, platelet; ALB, albumin; TB, total bilirubin; ALT, alanine transaminase; AST, aspartate aminotransferase; GGT, gamma-glutamyl transpeptidase; HB, hemoglobin.

### Survival Outcomes

The median RFS of all patients in this study was 12.1 months, and the median OS was 23.0 months. One-year, 2-year, and 3-year RFS rates were 51.3%, 29.6%, and 23.4%, respectively. The corresponding rates of OS were 65.4%, 49.5%, and 42.4%, respectively. Survival rates were significantly different between PVTT 0-2 and PVTT 3-4. The median RFS was 6.3 months for patients with PVTT 0-2 and 3.5 months for patients with PVTT 3-4 (*P*<0.001). The corresponding median OS was 32.9 months and 12.1 months, respectively (*P*<0.001).

### Survival Outcomes Development and Validation of a Prognostic Score

**Figure 1:**
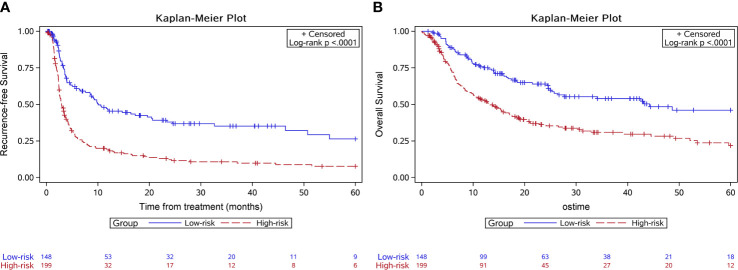
Kaplan-Meier survival curves of the survival outcome in the training group. The recurrence free survival **(A)** and the overall survival **(B)** were both significantly longer in the low-risk group.

**Figure 2:**
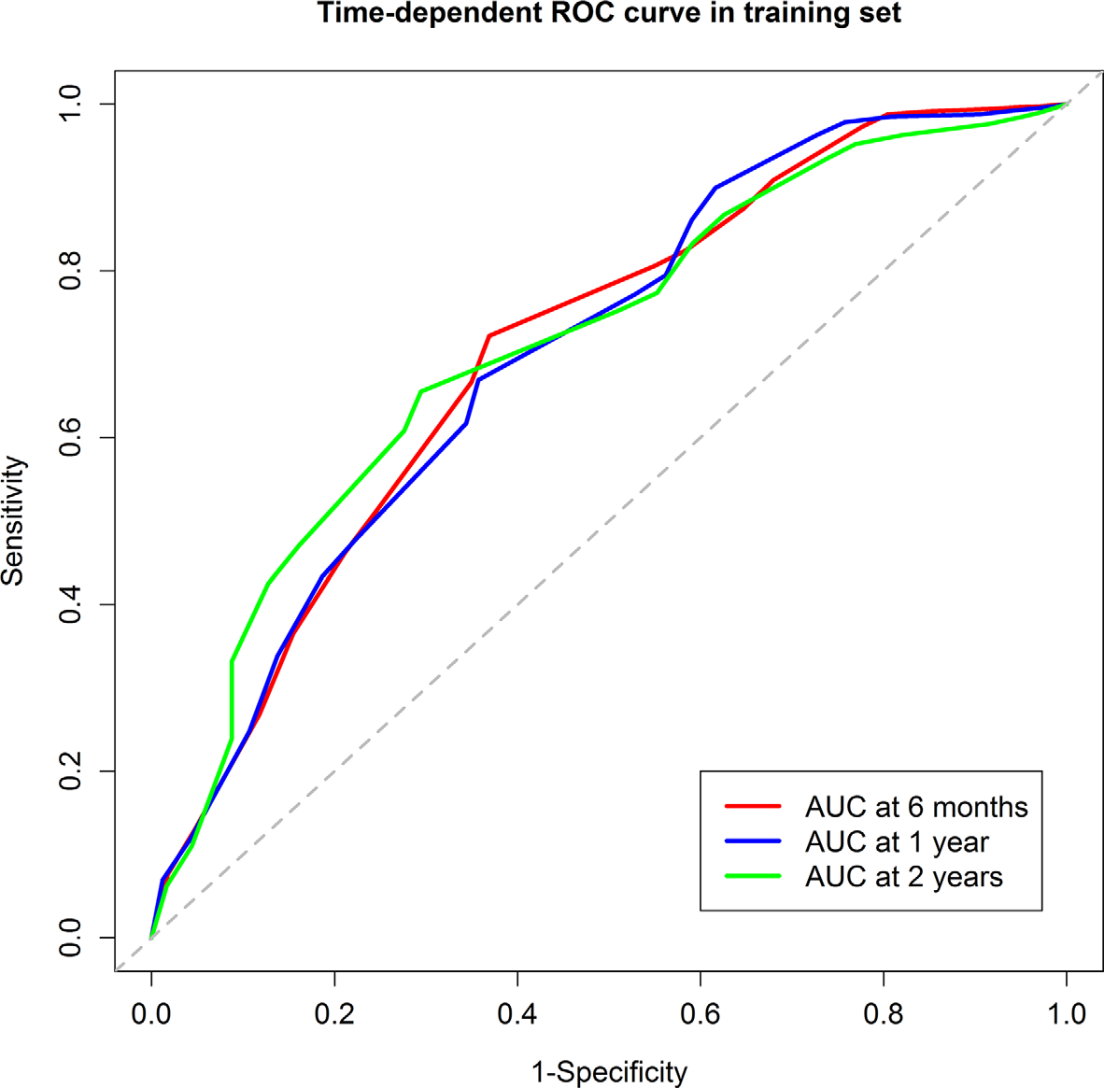
The Receiver Operating Characteristic curves of the 6-month, 1-year, and 2-year recurrence free survival of the training group. The areas under curve were 0.71, 0.70, and 0.71, respectively.

The score model was established based on the RFS data of the training group. Eventually, six factors were selected, which were the ALBI score (26), tumor size, the number of invaded liver segments, GGT, AFP, and the PVTT stage. The mark sheet was presented in **Table 2**. The score for each factor was determined by the coefficient in the stepwise Cox regression. Cutoff of the prognostic model was set to be 14, which could achieve the best AUC of the ROC curves. Patients in the training group were then divided into the low-risk group (score<14, n=148) and the high-risk group (score≥14, n=199). Median RFS of the low-risk group was significantly longer than that of the high-risk group (10.1 vs 2.9 months, *P*<0.001) (**Figure 1A**). The 6-month, 1-year, and 2-year RFS rates were 60.7%, 46.3%, and 38.0% for the low-risk group while the corresponding rates were only 27.4%, 19.4%, and 12.2% in the high-risk group, respectively. ROC curves for 6-month, 1-year, and 2-year RFS rates were presented in **Figure 2**. The corresponding AUCs were 0.71, 0.70, and 0.71 in the training group. Median OS was 44.3 months in the low-risk group, and only 13.1 months in the high-risk group (*P*<0.001) (**Figure 1B**).

**Table 2:** The mark sheet of pre-operative score.

Variable	Score
ALBI score>-2.6	3
Tumor size >5cm	4
Tumor invaded liver segments ≥3	2
GGT >50U/L	4
AFP >200ng/mL	3
PVTT stage 3-4	3

Low-risk: sum of the score less than 14; High-risk: sum of the score no less than 14.

GGT, gamma-glutamyl transpeptidase; AFP, alpha fetoprotein.

**Figure 3:**
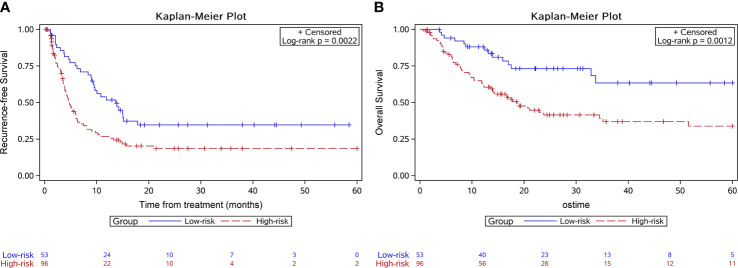
Kaplan-Meier survival curves of the survival outcome in the validation group. The recurrence free survival **(A)** and the overall survival **(B)** were both significantly longer in the low-risk group.

**Figure 4:**
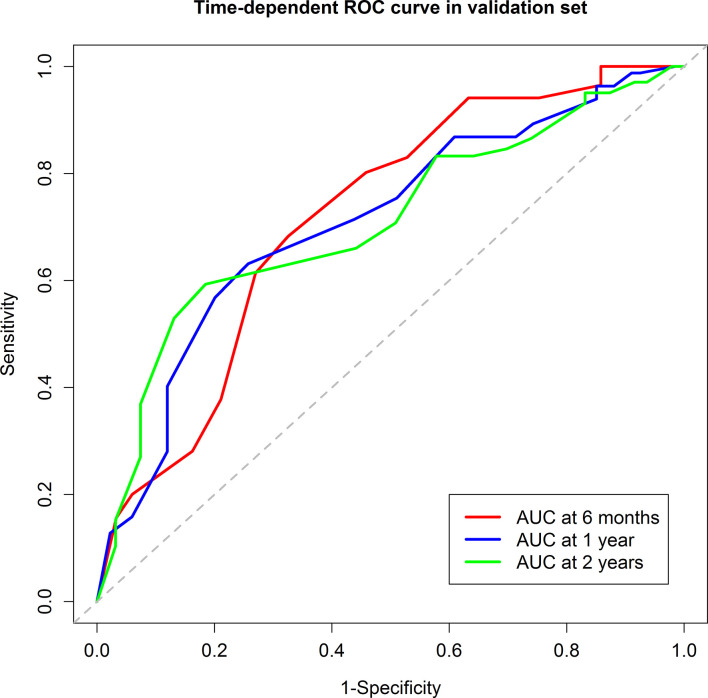
The Receiver Operating Characteristic curves of the 6-month, 1-year, and 2-year recurrence free survival of the validation group. The areas under curve were 0.72, 0.71, and 0.71, respectively.

In the validation group, 53 patients were in the low-risk group while 96 were in the high-risk group. Median RFS was 13.7 months for the low-risk group and 4.6 months for the high-risk group (*P*=0.002) (**Figure 3A**). The 6-month, 1-year, and 2-year RFS rates were 75.1%, 51.8%, and 34.7% for the low-risk group. The corresponding rates were 40.2%, 26.8%, and 18.5% in the high-risk group, respectively. ROC curves for 6-month, 1-year, and 2-year RFS rates were presented in **Figure 4**. The corresponding AUCs were 0.72, 0.71, and 0.71 in the validation group. Median OS was not reached for the low-risk group but was 18.7 months for the high-risk group (*P*=0.001) (**Figure 3B**).

Subgroup analysis showed that, for patients with ascites, PHT, thrombocytopenia, or in Child-Pugh class B (128 out of 497), the RFS of the low-risk group was significantly higher than that of the high-risk group (10.0 vs. 3.3 months, *P*<0.001). Results were similar in patients without these conditions (RFS in low-risk group vs. high-risk group: 11.6 vs. 3.6 months, *P*<0.001).

## Discussion

In this study, we established a pre-operative prognostic score for advanced HCC patients to select an appropriate population at the advanced stage who could gain survival benefit from surgery. The score was easy to obtain with pre-operative clinical data and was well validated.

Nowadays, systemic treatments are still the standard treatment for advanced stage HCC patients. Yet advanced HCC patients who undergo standard treatment are expected to have a median survival of only 8-13.6 months (4, 5, 27). In this article, advanced stage HCC patients who received resection could reach a median OS of 23.0 months, with 1-year and 3-year OS rates of 65.4% and 42.4%. These results are similar to a previous high-quality meta-analysis, with 1-year and 3-year OS rates of 62% and 42% for BCLC stage C patients (28). However, the survival result in our center is better than that reported in the EHBH-PVTT study (19), which was only 17.0 months even in the low-risk group. This might be due to the fact that the EHBH-PVTT study included more patients with PVTT 3 and mixed all PVTT stages together. However, survival outcomes could be distinct for patients with different PVTT stages (9). According to data in our center, patients with PVTT 2 had a median OS of 24.5 months, compared to only 10.6 months for patients with PVTT 3 (*P*=0.014). Neglection of the PVTT stages might lead to inadequate evaluation of survival benefit.

To our knowledge, this article is the first to establish a prognostic score on the basis of PVTT stage to help select a group of advanced HCC patients receiving surgery with a promising survival benefit. The score was based on pre-treatment clinical data so that patients could be divided into a high-risk group and a low-risk group before treatment selection. The median RFS of patients in the low-risk group was 10.9 months, and the median OS could reach 44.3 months, which was quite close to the OS of patients receiving resection in BCLC stage B in previous studies (29–31). The subgroup analysis of the phase III RCT of Sorafenib showed that no subgroup could achieve an OS longer than 15 months (32). Local therapy, such as transarterial chemoembolization (TACE), could achieve a median survival ranged from 13 to 35 months for non-TACE refractory patients (33). Survival for TACE-refractory patients was only 7 to 10.5 months. Despite the lack of direct comparison in this study, the survival advantage of resection in the low-risk group seemed to be superior to non-surgical treatments. The main cause of this promising result might be that resection has an obvious advantage in reducing tumor burden. Patients in the low-risk group tend to have better liver function, smaller tumors, and PVTT within the second order or more peripheral branch, which means anatomic resection (AR) is easier to achieve. AR is capable of eradicating potential micrometastases surrounding tumors (34), and was proven to be capable of providing better survival than non-AR (35). This might also contribute to the significant survival superiority of the low-risk group. Therefore, we recommend resection for advanced HCC patients who are estimated as low risk according to our score. On the other hand, the median OS for the high-risk group was 13.1 months, and the median RFS was only 2.9 months. One of the determining factors for the poor outcomes might be that more patients in the high-risk group had PVTT in the main trunk or the first-order branch. A previous study reported that the median survival was 0.91 years for patients with PVTT 4 and 1.58 years for patients with PVTT 3 (9). Radical removal of tumor thrombosis was difficult for these patients, which might cause an early recurrence. Therefore, resection should be carefully performed due to the limited survival and higher probability of severe complication in these patients. Prognosis of other treatments for these patients were incompetent as well. Jeong et al. investigated the efficacy of Sorafenib in patients with PVTT 3-4 and the median OS was only 3.1 months (36). TACE could achieve a median survival of 6.1 to 7.49 months, yet this is still unsatisfactory (37, 38). Further studies on other treatment strategies for this population are urgently needed. In our study, although the RFS were significantly different in the two groups, the median RFS was only 10.9 months even in the low-risk group. Although long-term survival could be achieved in selected advanced HCC patients, the recurrence rate was still quite high after resection. Evaluation of adjuvant and neoadjuvant therapies might be helpful to prolong survival for this population.

The composition of this score covers the three aspects of liver function, extent of the surgery, and tumor burden, which are also elements physician would consider clinically. The ALBI score and the level of GGT represent the liver function of the patient. The ALBI score involving bilirubin and albumin level was constructed in 2015 and was well validated in HCC patients (26, 39). The previous published EHBH-PVTT score selected bilirubin as the aspect of liver function. We considered our score might reflect liver function better. The number of tumor-invaded liver segments is the decisive factor of the planned extent of surgery and was only included in our score system. As for the tumor burden, most of the elements were similar in the two scores, except that we used the stage of PVTT as one of the prognostic factors. As mentioned above, survival differed a lot within different stages of PVTT. The EHBH cohort also concluded that stages of PVTT was associated with disease-free survival, yet this factor was not used for score construction. This might be due to the fact that the EHBH score used prognostic factors for OS while our score used factors for RFS. OS was largely affected by the status and treatment of recurrent tumors, which might cause bias in the retrospective analysis. Therefore, we considered that the construction of a prognostic score is better if based on RFS.

It is interesting that with a cutoff value of 14 in our score system, patients with poor liver function were all in the high-risk group even with smaller tumors, less invaded liver segments, lower AFP, and no PVTT in the main trunk or first-branches. Things were quite different in the prognostic system in early stages, where the tumor characteristics accounted for the majority of the prognostic model (40, 41). It seems that for patients in the advanced stage, liver function is the main limiting factor for long-term survival. Performance of surgery in patients with poor liver function should be considered with caution in the advanced stage.

There exist some limitations to this article. First, the approach of resection was not discussed in this article. Several studies showed that the approach of resection affected survival after surgery (42, 43). However, most patients in the advanced stage would receive open abdominal surgery for the complexity of the disease. Therefore, this might not affect the practicability of the prognostic score. Second, we did not make a comparison of resection to the standard treatment of advanced HCC in the two risk groups. This is because the proportion of patients receiving Sorafenib is quite low due to the limited cost-effectiveness. Further studies are needed for this comparison. Third, it should be noted that several conditions were not included during the development of the score, such as patients without preserved liver function (Child-Pugh score>8) and patients with extrahepatic metastasis. Clinically, curative resection on these patients were considered extremely difficult or might cause severe post-surgery complications. Evaluation on these patients was not practical. Therefore, this article only discussed long-term OS in patients who were evaluated as resectable clinically. Last, this study was conducted based on a single center cohort in China. Therefore, the generalizability of our results is limited in western populations and needs further validation.

In conclusion, we established an effective pre-operative score that could help to select a group of advanced HCC patients who might benefit from liver resection with promising long-term survival. We recommended resection for patients in the low-risk group.

## Data Availability Statement

The raw data supporting the conclusions of this article will be made available by the authors, without undue reservation.

## Ethics Statement

The studies involving human participants were reviewed and approved by the ethics committee of the First Affiliated Hospital of Sun Yat-sen University. Written informed consent for participation was not required for this study in accordance with the national legislation and the institutional requirements.

## Author Contributions

Concept and design: MK, SP, HX, and S-LC. Data collection: HX, J-LL, M-MT, Z-WP, and S-TF. Acquisition, analysis, or interpretation of data: All authors. Drafting of the manuscript: HX, S-LC, SP, and MK. Critical revision of the manuscript for important intellectual content: SP, S-LC, and MK. Statistical analysis: QZ, T-FW, and XL. Supervision: MK and SP. All authors contributed to the article and approved the submitted version.

## Funding

This work is supported by grants from the National Natural Science Foundation of China (NSFC, No. 81770608, 81801703), the National Science Fund for Distinguished Young Scholars (No. 81825013), the Natural Science Foundation of Guangdong Province (No. 2018A030310282), the Kelin Outstanding Young Scientist of the First Affiliated Hospital of Sun Yet-sen University (2017), and the Guangdong Basic and Applied Basic Research Foundation (No. 2019A1515111168).

## Conflict of Interest

Authors T-FW and XL was employed by GE Healthcare.

The remaining authors declare that the research was conducted in the absence of any commercial or financial relationships that could be construed as a potential conflict of interest.

## References

[1] Siegel RL Miller KD Jemal A. Cancer statistics, 2018. CA Cancer J Clin (2018) 68:7–30. 10.3322/caac.21442 29313949

[2] Chen W Sun K Zheng R Zeng H Zhang S Xia C. Cancer incidence and mortality in China, 2014. Chin J Cancer Res (2018) 30:1–12. 10.21147/j.issn.1000-9604.2018.01.01 29545714 PMC5842223

[3] Park J-W Chen M Colombo M Roberts LR Schwartz M Chen P-J. Global patterns of hepatocellular carcinoma management from diagnosis to death: the BRIDGE Study. Liver Int (2015) 35:2155–66. 10.1111/liv.12818 PMC4691343 25752327

[4] Llovet JM Ricci S Mazzaferro V Hilgard P Gane E Blanc J-F. Sorafenib in advanced hepatocellular carcinoma. N Engl J Med (2008) 359:378–90. 10.1056/NEJMoa0708857 18650514

[5] Kudo M Finn RS Qin S Han K-H Ikeda K Piscaglia F. Lenvatinib versus sorafenib in first-line treatment of patients with unresectable hepatocellular carcinoma: a randomised phase 3 non-inferiority trial. Lancet (2018) 391:1163–73. 10.1016/S0140-6736(18)30207-1 29433850

[6] Roayaie S Jibara G Tabrizian P Park J-W Yang J Yan L. The role of hepatic resection in the treatment of hepatocellular cancer. Hepatology (2015) 62:440–51. 10.1002/hep.27745 25678263

[7] Zhong J-H Ke Y Gong W-F Xiang B-D Ma L Ye X-P. Hepatic resection associated with good survival for selected patients with intermediate and advanced-stage hepatocellular carcinoma. Ann Surg (2014) 260:329–40. 10.1097/SLA.0000000000000236 24096763

[8] Ruzzenente A Capra F Pachera S Iacono C Piccirillo G Lunardi M. Is liver resection justified in advanced hepatocellular carcinoma? Results of an observational study in 464 patients. J Gastrointest Surg (2009) 13:1313–20. 10.1007/s11605-009-0903-x 19418103

[9] Kokudo T Hasegawa K Matsuyama Y Takayama T Izumi N Kadoya M. Survival benefit of liver resection for hepatocellular carcinoma associated with portal vein invasion. J Hepatol (2016) 65:938–43. 10.1016/j.jhep.2016.05.044 27266618

[10] Heimbach JK Kulik LM Finn RS Sirlin CB Abecassis MM Roberts LR. AASLD guidelines for the treatment of hepatocellular carcinoma. Hepatology (2017) 67:358–80. 10.1002/hep.29086 28130846

[11] European Association for the Study of the Liver. Electronic address: easloffice@easloffice.eu, European Association For The Study Of The Liver. EASL Clinical Practice Guidelines: Management of hepatocellular carcinoma. J Hepatol (2018) 69:182–236. 10.1016/j.jhep.2018.03.019 29628281

[12] Omata M Cheng A-L Kokudo N Kudo M Lee JM Jia J. Asia-Pacific clinical practice guidelines on the management of hepatocellular carcinoma: a 2017 update. Hepatol Int (2017) 11:317–70. 10.1007/s12072-017-9799-9 PMC5491694 28620797

[13] Kokudo N Hasegawa K Akahane M Igaki H Izumi N Ichida T. Evidence-based Clinical Practice Guidelines for Hepatocellular Carcinoma: The Japan Society of Hepatology 2013 update (3rd JSH-HCC Guidelines). Hepatol Res (2015) 45:123–7. 10.1111/hepr.12464 25625806

[14] Chok KSH Cheung TT Chan SC Poon RTP Fan ST Lo CM. Surgical outcomes in hepatocellular carcinoma patients with portal vein tumor thrombosis. World J Surg (2014) 38:490–6. 10.1007/s00268-013-2290-4 24132826

[15] Xu J-F Liu X-Y Wang S Wen H-X. Surgical treatment for hepatocellular carcinoma with portal vein tumor thrombus: a novel classification. World J Surg Oncol (2015) 13:86. 10.1186/s12957-015-0493-x 25889711 PMC4352541

[16] Peng Z-W Guo R-P Zhang Y-J Lin X-J Chen M-S Lau WY. Hepatic resection versus transcatheter arterial chemoembolization for the treatment of hepatocellular carcinoma with portal vein tumor thrombus. Cancer (2012) 118:4725–36. 10.1002/cncr.26561 22359112

[17] Matono R Yoshiya S Motomura T Toshima T Kayashima H Masuda T. Factors linked to longterm survival of patients with hepatocellular carcinoma accompanied by tumour thrombus in the major portal vein after surgical resection. HPB (Oxford) (2012) 14:247–53. 10.1111/j.1477-2574.2011.00436.x PMC3371211 22404263

[18] Kokudo T Hasegawa K Matsuyama Y Takayama T Izumi N Kadoya M. Liver resection for hepatocellular carcinoma associated with hepatic vein invasion: A Japanese nationwide survey. Hepatology (2017) 66:510–7. 10.1002/hep.29225 28437844

[19] Zhang X-P Gao Y-Z Chen Z-H Chen M-S Li L-Q Wen T-F. An Eastern Hepatobiliary Surgery Hospital/Portal Vein Tumor Thrombus scoring system as an aid to decision-making on hepatectomy for Hepatocellular Carcinoma patients with Portal Vein Tumor Thrombus: a multicenter study. Hepatology (2018) 692076–90. 10.1002/hep.30490 30586158

[20] European Association for Study of Liver, European Organisation for Research and Treatment of Cancer. EASL-EORTC clinical practice guidelines: management of hepatocellular carcinoma. Eur J Cancer (2012) 48:599–641. 10.1016/j.ejca.2011.12.021 22424278

[21] Bruix J Sherman M Llovet JM Beaugrand M Lencioni R Burroughs AK. Clinical management of hepatocellular carcinoma. In: Conclusions of the barcelona-2000 EASL conference J Hepatol 35:421–30. 10.1016/S0168-8278(01)00130-1 11592607

[22] Bruix J Sherman M. Practice Guidelines Committee, American Association for the Study of Liver Diseases. Management of hepatocellular carcinoma. Hepatology (2005) 42:1208–36. 10.1002/hep.20933 16250051

[23] Bruix J Reig M Sherman M. Evidence-Based Diagnosis, Staging, and Treatment of Patients With Hepatocellular Carcinoma. Gastroenterology (2016) 150:835–53. 10.1053/j.gastro.2015.12.041 26795574

[24] Forner A Llovet JM Bruix J. Hepatocellular carcinoma. Lancet (2012) 379:1245–55. 10.1016/S0140-6736(11)61347-0 PMC13537732 22353262

[25] Lencioni R Llovet JM. Modified RECIST (mRECIST) assessment for hepatocellular carcinoma. Semin Liver Dis (2010) 30:52–60. 10.1055/s-0030-1247132 20175033 PMC12268942

[26] Johnson PJ Berhane S Kagebayashi C Satomura S Teng M Reeves HL. Assessment of liver function in patients with hepatocellular carcinoma: a new evidence-based approach-the ALBI grade. J Clin Oncol (2015) 33:550–8. 10.1200/JCO.2014.57.9151 PMC4322258 25512453

[27] Cheng A-L Kang Y-K Chen Z Tsao C-J Qin S Kim JS. Efficacy and safety of sorafenib in patients in the Asia-Pacific region with advanced hepatocellular carcinoma: a phase III randomised, double-blind, placebo-controlled trial. Lancet Oncol (2009) 10:25–34. 10.1016/S1470-2045(08)70285-7 19095497

[28] Hyun MH Lee Y-S Kim JH Lee CU Jung YK Seo YS. Hepatic resection compared to chemoembolization in intermediate- to advanced-stage hepatocellular carcinoma: A meta-analysis of high-quality studies. Hepatology (2018) 68:977–93. 10.1002/hep.29883 29543988

[29] Ng KK Vauthey J-N Pawlik TM Lauwers GY Regimbeau J-M Belghiti J. Is hepatic resection for large or multinodular hepatocellular carcinoma justified? Results from a multi-institutional database. Ann Surg Oncol (2005) 12:364–73. 10.1245/ASO.2005.06.004 15915370

[30] Kim H Ahn SW Hong SK Yoon KC Kim H-S Choi YR. Survival benefit of liver resection for Barcelona Clinic Liver Cancer stage B hepatocellular carcinoma. Br J Surg (2017) 104:1045–52. 10.1002/bjs.10541 28480964

[31] Wang B-W Mok K-T Liu S-I Chou N-H Tsai C-C Chen I-S. Is hepatectomy beneficial in the treatment of multinodular hepatocellular carcinoma? J Formos Med Assoc (2008) 107:616–26. 10.1016/S0929-6646(08)60179-5 18678545

[32] Bruix J Raoul J-L Sherman M Mazzaferro V Bolondi L Craxì A. Efficacy and safety of sorafenib in patients with advanced hepatocellular carcinoma: subanalyses of a phase III trial. J Hepatol. (2012) 57:821–9. 10.1016/j.jhep.2012.06.014 PMC12261288 22727733

[33] Kodama K Kawaoka T Aikata H Uchikawa S Inagaki Y Hatooka M. Comparison of clinical outcome of hepatic arterial infusion chemotherapy and sorafenib for advanced hepatocellular carcinoma according to macrovascular invasion and transcatheter arterial chemoembolization refractory status. J Gastroenterol Hepatol (2018) 33:1780–6. 10.1111/jgh.14152 29645345

[34] Makuuchi M Hasegawa H Yamazaki S. Ultrasonically guided subsegmentectomy. Surg Gynecol Obstet (1985) 161:346–50. 10.1055/s-2007-1022639 2996162

[35] Shindoh J Makuuchi M Matsuyama Y Mise Y Arita J Sakamoto Y. Complete removal of the tumor-bearing portal territory decreases local tumor recurrence and improves disease-specific survival of patients with hepatocellular carcinoma. J Hepatol (2016) 64:594–600. 10.1016/j.jhep.2015.10.015 26505120

[36] Jeong SW Jang JY Shim KY Lee SH Kim SG Cha S-W. Practical Effect of Sorafenib Monotherapy on Advanced Hepatocellular Carcinoma and Portal Vein Tumor Thrombosis. Gut Liver (2013) 7:696–703. 10.5009/gnl.2013.7.6.696 24312711 PMC3848540

[37] Zhao Y Duran R Chapiro J Sohn JH Sahu S Fleckenstein F. Transarterial Chemoembolization for the Treatment of Advanced-Stage Hepatocellular Carcinoma. J Gastrointest Surg (2016) 20:2002–9. 10.1007/s11605-016-3285-x PMC5106296 27714643

[38] Lv W-F Liu K-C Lu D Zhou C-Z Cheng D-L Xiao J-K. Transarterial chemoembolization for hepatocellular carcinoma combined with portal vein tumor thrombosis. CMAR (2018) 10:4719–26. 10.2147/CMAR.S166527 PMC6199972 30410405

[39] Kao W-Y Su C-W Chiou Y-Y Chiu N-C Liu C-A Fang K-C. Hepatocellular Carcinoma: Nomograms Based on the Albumin-Bilirubin Grade to Assess the Outcomes of Radiofrequency Ablation. Radiology (2017) 285:162382–680. 10.1148/radiol.2017162382 28562211

[40] Zheng J Chou JF Gonen M Vachharajani N Chapman WC Majella Doyle MB. Prediction of Hepatocellular Carcinoma Recurrence Beyond Milan Criteria After Resection: Validation of a Clinical Risk Score in an International Cohort. Ann Surg (2017) 266:693–701. 10.1097/SLA.0000000000002360 28650354 PMC8404085

[41] Cho CS Gonen M Shia J Kattan MW Klimstra DS Jarnagin WR. A novel prognostic nomogram is more accurate than conventional staging systems for predicting survival after resection of hepatocellular carcinoma. J Am Coll Surgeons (2008) 206:281–91. 10.1016/j.jamcollsurg.2007.07.031 18222381

[42] Lee JJ Conneely JB Smoot RL Gallinger S Greig PD Moulton C-A. Laparoscopic versus open liver resection for hepatocellular carcinoma at a North-American Centre: a 2-to-1 matched pair analysis. HPB (Oxford) (2015) 17:304–10. 10.1111/hpb.12342 PMC4368393 25297815

[43] Yoon Y-S Han H-S Cho JY Ahn KS. Total laparoscopic liver resection for hepatocellular carcinoma located in all segments of the liver. Surg Endosc (2010) 24:1630–7. 10.1007/s00464-009-0823-6 20035349

[44] Xiao H Chen S-L Tang M-M Peng Z-W Peng S Kuang M. A pre-operative prognostic score for patients with advanced hepatocellular carcinoma (HCC) who underwent resection. J Digest Dis (2019) 20:S1 38–39. 10.1111/1751-2980.12808 PMC7953908 33718130

